# MicroRNA Expression Profiling of Normal and Malignant Human Colonic Stem Cells Identifies *miRNA92a* as a Regulator of the *LRIG1* Stem Cell Gene

**DOI:** 10.3390/ijms21082804

**Published:** 2020-04-17

**Authors:** Vignesh Viswanathan, Lynn Opdenaker, Shirin Modarai, Jeremy Z. Fields, Gregory Gonye, Bruce M. Boman

**Affiliations:** 1 Center for Translational Cancer Research, Helen F Graham Cancer Center and Research Institute, Newark, DE 19713, USA; 2 Department of Biological Sciences, University of Delaware, Newark, DE 19711, USA; 3 CA*TX Inc., Princeton, NJ 08542, USA; 4 Nanostring Technologies, Seattle, WA 98109, USA; 5 Department of Pharmacology & Experimental Therapeutics, Thomas Jefferson University & Kimmel Cancer Center, Philadelphia, PA 19107, USA

**Keywords:** microRNA, *miRNA92a*, stem cells, *LRIG1*, colon, human, crypts

## Abstract

MicroRNAs (miRNAs) have a critical role in regulating stem cells (SCs) during development, and because aberrant expression of miRNAs occurs in various cancers, our goal was to determine if dysregulation of miRNAs is involved in the SC origin of colorectal cancer (CRC). We previously reported that aldehyde dehydrogenase (ALDH) is a marker for normal and malignant human colonic SCs and tracks SC overpopulation during colon tumorigenesis. MicroRNA expression was studied in ALDH-positive SCs from normal and malignant human colon tissues by Nanostring miRNA profiling. Our findings show that: (1) A unique miRNA signature distinguishes ALDH-positive CRC cells from ALDH-positive normal colonic epithelial cells, (2) Expression of four miRNAs (*miRNA200c*, *miRNA92a*, *miRNA20a*, *miRNA93*) are significantly altered in CRC SCs compared to normal colonic SCs, (3) *miRNA92a* expression is also upregulated in ALDH-positive HT29 CRC SCs as compared to ALDH-negative SCs, (4) *miRNA92a* targets the 3′UTR of *LRIG1* SC gene, and (5) *miRNA92a* modulates proliferation of HT29 CRC cells. Thus, our findings indicate that overexpression of *miRNA92a* contributes to the SC origin of CRC. Strategies designed to modulate miRNA expression, such as *miRNA92a*, may provide ways to target malignant SCs and to develop more effective therapies against CRC.

## 1. Introduction

A significant body of scientific evidence indicates that: (i) stem cells (SCs) are the cells of origin of cancer [1,2], (ii) SC overpopulation drives tumor initiation and progression [3,4,5], (iii) SCs are resistant to conventional anti-cancer therapies. We discovered that aldehyde dehydrogenase (ALDH) is a marker for normal and malignant human colonic SCs and tracks SC overpopulation during colon tumorigenesis [5]. While our findings on ALDH and many results on various SC markers by others indicates that cancer SC (CSC) overpopulation drives tumor growth, it is not fully understood which dysregulated mechanisms cause the SC overpopulation. Because evidence indicates miRNAs have an important role in the pathogenesis of various cancers, we have been studying changes in miRNAs as a possible mechanism in colorectal cancer (CRC). Indeed, aberrant expression of miRNAs leads to wide-spread transcriptional dysregulation and cancer [6,7,8,9,10]. In CRC, differential miRNA expression has been related to stage and site of the tumor [11]. Mounting evidence also indicates a role for miRNAs in the maintenance of the CSC phenotype [12,13,14,15,16]. We have been focusing on miRNAs that contribute to maintenance of the phenotype of colonic CSCs.

Indeed, a previous study of ours was designed to analyze miRNA expression in the colonic SC niche using micro-dissected human colonic crypts. In that study we identified a set of miRNAs that is specific to the normal colonic SC niche and distinguishes malignant from normal epithelia [17,18]. Notably, *miRNA23b*, which was increased in CRC, was predicted to target the SC-expressed G protein-coupled receptor LGR5. Further investigations showed that *miRNA23b* regulates CSC phenotypes globally at the level of proliferation, cell-cycle, self-renewal, EMT, invasion, and resistance to the CRC chemotherapeutic agent 5-FU. We also found that *miRNA23b* decreased LGR5 expression and increased the number of ALDH-positive CSCs. CSC analyses confirmed that levels of LGR5 and *miRNA23b* are inversely correlated in ALDH-positive CSCs and that CRC tissues contain distinct sub-populations of LGR5-positive and ALDH-positive CSCs. Overall, our previous study defined a critical function for *miRNA23b*, which, by targeting LGR5, contributes to overpopulation of ALDH-positive CSCs and CRC.

In the current study, we took a different approach to identify miRNAs in colonic SCs based on a more extensive screen that involved miRNA expression profiling of ALDH-positive SCs isolated from normal colonic epithelium and CRC tissue. This broader approach was done to identify additional miRNAs that might be involved in the SC origin of CRC development. Accordingly, we used nCounter miRNA profiling (Nanostring Technologies) to analyze miRNA expression in purified ALDH-positive cells from human CRCs and normal colonic epithelium (NCE). We hypothesized that specific miRNAs are selectively expressed in the normal ALDH-positive SCs, and that some or all of these miRNAs contribute to SC mechanisms involved in CRC growth during tumor development. Our initial goal was to identify the set of miRNAs and their target genes that are specific to the normal colonic ALDH-positive SCs. Our second goal was to identify specific miRNAs in that set that are aberrantly expressed in CRC ALDH-positive CSCs. Our third goal was to see if some of these miRNAs are predicted to target known SC marker genes, and whether they contribute to the SC origin of CRC.

## 2. Results

### 2.1. Identification and Isolation of ALDH-Positive Normal and Malignant Human Colonic Stem Cells

**Figure 1:**
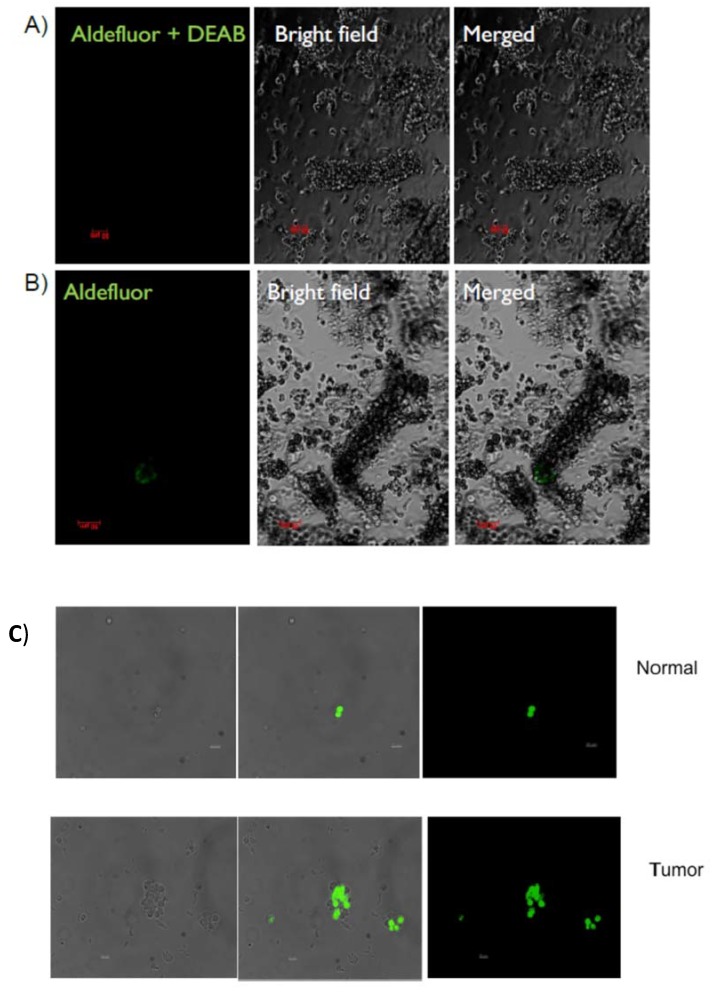

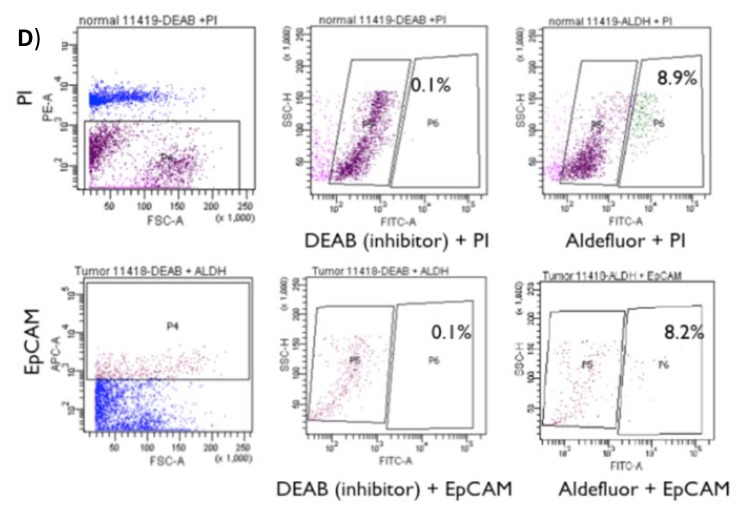
Identification and isolation of ALDEFLUOR-positive stem cells from fresh patient normal and tumor samples. Panels A, B—ALDH activity in the bottom of fresh normal isolated crypts. Normal isolated colonic crypts subjected to ALDEFLUOR assay in the presence (**A**) or absence of the inhibitor of ALDH activity (**B**). This image was taken using a Zeiss Epi-fluorescence microscope using the 10X objective. (**C**) Panel C shows ALDH activity in fresh dissociated patient tumor cells. Dissociated cells from fresh normal and tumor tissue show small populations of ALDEFLUOR positive (green) cells. Image was taken using a Zeiss Epi-fluorescence microscope using the 10X objective. (**D**) Panel D shows a histogram for flow cytometric isolation of stem cells from fresh patient normal and tumor samples. Gates showing representative percentages of isolated cells from fresh normal and tumor tissue positive for ALDH activity, when the DEAB control was set to 0.1%. Tumor cells were selected for EpCAM positivity (carcinoma cells) and normal cells negative for Propidium iodide (viable cells) for ALDEFLUOR assay and sorting.

To identify ALDH-positive SCs in viable human tissues, isolated human colonic crypts as well as freshly dissociated normal and malignant colonic cells, were subjected to the ALDEFLUOR assay in the presence or absence of the DEAB inhibitor of ALDH activity. Fluorescence microscopy analysis showed that cells at the bottom of normal colonic crypts display intense green fluorescence signal indicating that cells in the crypt SC niche have high ALDH activity (Figure 1A,B). Fluorescence microscopic analysis of dissociated cells derived from both fresh normal crypts and CRC tissues also showed that these colonic tissues contain specific subpopulations of ALDEFLUOR-positive (green stained) cells (Figure 1C). These results indicate that high ALDH activity specifically marks colonic SCs in fresh viable normal and malignant colonic tissues. Flow cytometric cell sorting was then used to isolate SCs from fresh normal and tumor patient samples. Cells that co-stained for both ALDEFLUOR and EpCAM (for marking carcinoma cells) were sorted along with those that were EpCAM-positive and ALDEFLUOR-negative. ALDEFLUOR-positive and negative cells from five pairs of matched normal and tumor tissues were sorted (Figure 1D). The proportion of ALDEFLUOR-positive cells in the different cell populations isolated from patient samples are given in Supplementary Table S1, which represents cells that were purified for miRNA expression analysis. For normal colon, the percent ALDEFLUOR-positive cells (3.8 ± 2.2%) from flow cytometry concurs with the percent (5.6%) reported by quantitative immunohistochemical mapping [5]. For colon carcinomas, the number of ALDEFLUOR-positive cells (~2%) in any given malignancy (10^9^–10^13^ cells) indicates that a large increase in absolute number of CSCs occurs during tumor development.

### 2.2. Differential miRNA Expression in ALDH-Positive CRC Stem Cells Compared to the Normal Stem Cells

Flow-sorted samples were then analyzed further by Nanostring-based profiling to identify miRNAs that are differentially expressed in tumor ALDEFLUOR-positive cells compared to normal ALDEFLUOR-positive cells. Nanostring profiling showed that a set of miRNAs are selectively expressed in normal colonic ALDEFLUOR-positive SCs (Supplementary Table S2). The heatmap in Supplementary Figure S1 shows an example (heatmap) of the differential expression of all miRNAs surveyed by Nanostring profiling (n = 800) from a representative experiment involving the four sorted cell populations isolated from matched fresh normal and tumor tissue from one patient.

**Figure 2:**
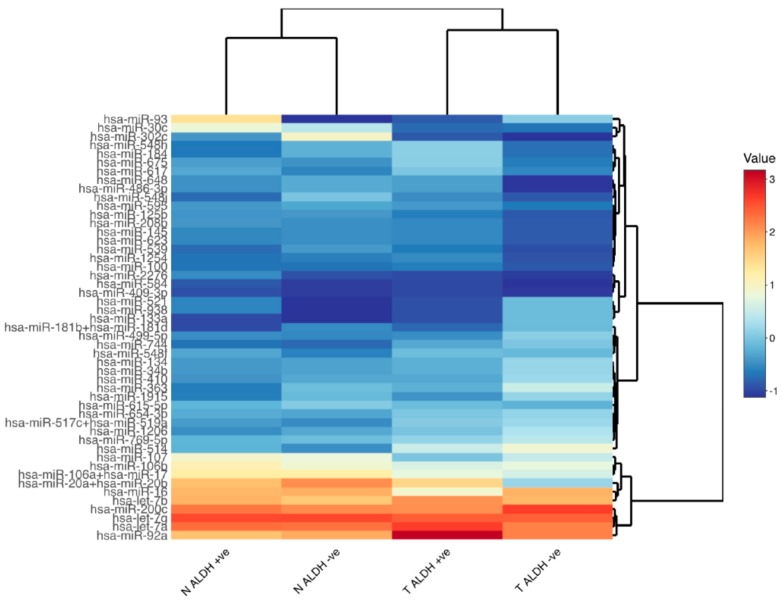
Differential expression of microRNAs in normal and tumor ALDEFLUOR positive and negative cells. This figure shows a focused heatmap for the subset of miRNAs based on statistical analysis (cutoff of *p* < 0.1) of all patient cases assessed by Nanostring profiling. The results are expressed as the average of normalized counts for the four types of sorted cell samples, (ALDH-positive and -negative cells for normal (N) and tumor (T)), which is converted to log2 and scaled to the mean of each sample. The list of differentially expressed miRNAs shown in Figure 2 is given in Supplementary Table S2.

To identify candidate miRNAs, we analyzed profiling results from multiple patient samples. We first focused on selecting miRNAs that showed a substantial difference in expression across multiple experiments and multiple patients. Specifically, this analysis provided the combined pattern of miRNA expression across the profiling of RNA from all four different isolated cell populations from all of the patients (n = 4). This analysis produced a top set of miRNAs having substantial differential expression (*p* < 0.1) in ALDEFLUOR-positive CSCs. These results (Figure 2) are displayed as a heatmap, which represents each miRNA’s relative log 2-fold change from the mean of the sample across all samples from multiple patients.

We then selected those miRNAs from this set that showed a statistically significant difference (*p* < 0.05) in expression in tumor CSCs compared to normal SCs. Specifically, our miRNA profiling identified significantly altered expression (*p* < 0.05) of *miRNA200c*, *miRNA92a*, *miRNA20a* and *miRNA93* in ALDEFLUOR-positive tumor CSCs as compared to ALDEFLUOR-positive normal SCs (Supplementary Figure S2). This sub-set of miRNAs was analyzed further to identify SC marker genes that are targeted by those miRNAs that are differentially expressed in tumor ALDEFLUOR-positive stem cells. Accordingly, miRNA target prediction tools (rna22 and TARGETSCAN) were used to see if any of the miRNAs are predicted to target known colonic SC markers. This analysis revealed that *miRNA92a* is predicted to target the 3′ UTR of the *LRIG1* SC gene. Thus, we selected *miRNA92a* for further analysis as described below.

### 2.3. miRNA92a Shows Differential Expression in ALDEFLUOR-Positive Cancer Stem Cells and Targets the LRIG1 Stem Cell Marker Gene

**Figure 3:**
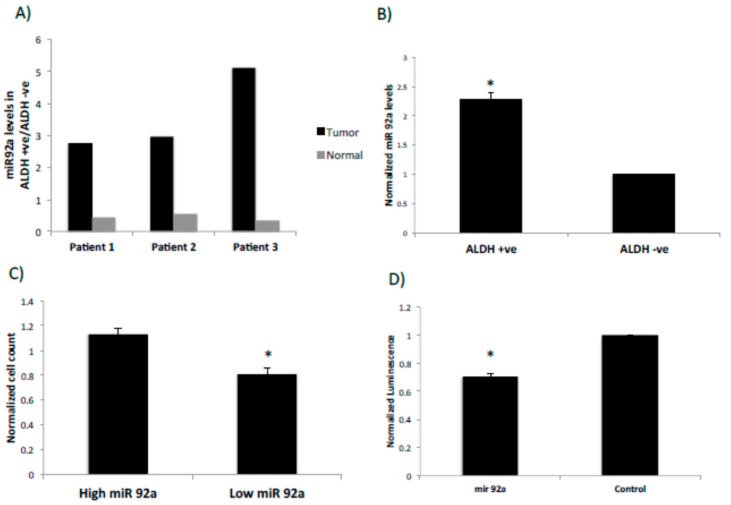
*MicroRNA92a* is overexpressed in ALDEFLUOR positive cells and regulates the *LRIG1* gene expression. (**A**) *MicroRNA92a* expression in tumor and normal ALDEFLUOR positive cells compared to ALDEFLUOR negative cells in patient samples. The results show *miRNA92a* expression is upregulated in ALDH-positive SCs from CRCs compared to ALDH-positive SCs from normal colonic epithelium. (**B**) Normalized *miRNA92a* expression levels in sorted ALDEFLUOR positive and negative HT29 cells. The results show *miRNA92a* expression is upregulated in ALDH-positive cells compared to ALDH-negative cells from the HT29 CRC line. (**C**) Normalized fold change in cell count of HT29 cells with increased and decreased levels of *miRNA92a*. The results show transfecting HT29 cells with *miRNA92a* antimir significantly reduces cell numbers and *miRNA92a* precursor has the opposite effect. (**D**) Luciferase assay shows that *miRNA92a* targets 3′UTR of *LRIG1* gene indicated by the significant decrease in the relative luminescence intensity as compared to the control. The results indicate *miRNA92a* down-modulates LRIG expression. Error bars represent standard error of mean and * represents a significant *p* value < 0.05.

We identified multiple differentially expressed miRNAs in colon cancers that have also been investigated and reported for having a role in cancer stem cells [19,20,21]. We decided to focus our attention on the miRNAs of 17–92 cluster [22], particularly *miRNA92a*, which has not been extensively investigated in the context of regulation of cancer stem cells. To evaluate a role of *miRNA92a* in colonic SCs, expression of *miRNA92a* was further analyzed in ALDEFLUOR-positive and negative SCs from fresh human colonic tissues and from the HT29 CRC cell line. Results show that *miRNA92a* expression is up-regulated in ALDEFLUOR-positive tumor cells compared to ALDEFLUOR-positive normal colonic cells from patient samples (Figure 3A). Further analysis of the HT29 cell line showed that *miRNA92a* expression is significantly upregulated in ALDEFLUOR-positive cells as compared to ALDEFLUOR-negative HT29 cells (Figure 3B). A proliferation assay was then done to assess the effect of this miRNA on the growth of colon cancer cells. Cell growth analysis showed that transfection with *miRNA92a* antimir significantly reduces proliferation of HT29 CRC cells and transfection with *miRNA92a* precursor siRNA has the opposite effect on proliferation (Figure 3C).

Because miRNA target prediction tools revealed that *miRNA92a* targets the *LRIG1* SC gene, we used luciferase assay to validate this target prediction. This luciferase assay employed a plasmid reporter that expresses luciferase and a 3′UTR of the predicted target *LRIG1* gene. This assay revealed a significant decrease in the relative luminescence intensity in transfected HT29 cells as compared to the control providing evidence that *miRNA92a* targets the *LRIG1* 3′UTR (Figure 3D).

## 3. Discussion

The main findings of our study are: (1) a set of miRNAs are selectively expressed in normal colonic ALDH-positive SCs, (2) specific miRNAs in this set are aberrantly expressed in CRC CSCs, (3) one of these aberrantly expressed miRNAs, *miRNA92a*, targets the 3′UTR of the *LRIG1* SC gene, and (4) *miRNA92a* regulates proliferation of human CRC cells. Our results support our hypothesis that specific miRNAs are selectively expressed in normal ALDH-positive SCs, and that some or all of these miRNAs contribute to SC mechanisms involved in CRC growth and development. Several points related to these findings bear further discussion.

### 3.1. What Is the Biological Significance of Differentially Expressed miRNAs in ALDH-Positive Colonic SCs?

There were several miRNAs that showed a differential expression in ALDH positive cells of the tumor compared to the ALDH positive cells of matched normal tissue. These findings suggest that dysregulation of these miRNAs results in transformation of normal SCs into tumor SCs. Our profiling results identified some members, particularly *miRNA200c*, of the miRNA200 family of miRNAs that are aberrantly expressed in CRC CSCs. The miRNA200 family consists of 5 members which have been shown to play key roles in cancer initiation and metastasis. For example, Shimono et al., reported that downregulation of *miRNA-200c* links breast cancer SCs with normal stem cells [19]. Moreover, we found some Let-7 family members were differentially expressed in tumor ALDH-positive cells. Let-7 was one of the first miRNAs shown to have a role in breast cancer initiating cells. It was downregulated in breast CSCs and induction of Let-7 expression inhibited their self-renewal [23]. *MicroRNA93* is an interesting candidate miRNA because it is downregulated in tumor SCs compared to normal SCs. *MicroRNA93* expression has also been reported to be significantly decreased in colon tumors compared to the normal counterpart and has been associated with stage and outcome of the tumor [20]. In breast cancer cells, increased expression of *miRNA93* induced MET (reverse of EMT), downregulated multiple SC-associated genes, and depleted CSC populations [21]. Moreover, in the colon cancer cell line SW116, *miRNA93* was found to be downregulated and then when upregulated, it was shown to suppress proliferation and colony formation [24].

We also showed that *miRNA92a* and *miRNA20a* are differentially expressed in patient-derived CSCs. Both these miRNAs are a part of the oncogenic miRNA17–92 cluster [24,25,26,27], which was predicted to target *LRIG1* [28,29]. The miRNA 17–92 cluster is upregulated in various forms of cancers such as hepatocellular and gastric carcinomas [30,31]. Moreover, members of this cluster have been reported to have a role in maintaining self-renewal of gastric CSCs by modulating levels of inhibitors of the WNT signaling pathway [31]. Moreover, *miRNA20a* was found to be overexpressed in prostate cancer and to be involved in metastasis and invasion [32]. High expression levels of the miRNA17-92 cluster have also been reported to have prognostic value in CRC [33]. Indeed, our miRNA profiling revealed that both *miRNA20a* and *miRNA92a* have significantly altered expression in ALDEFLUOR-positive tumor cells as compared to ALDEFLUOR-positive normal cells. However, our data showed that only *miRNA92a* was differentially expressed in the ALDELFUOR-positive HT29 CRC cells. Thus, among the differentially expressed miRNAs that we found in ALDH-positive colonic CSCs, *miRNA92a* appeared to have the most biological relevance in CRC.

### 3.2. What Is the Biological Significance of miRNA92a in Carcinogenesis?

Our further analysis of *miRNA92a* showed that it regulates proliferation of human CRC cells. Similarly, *miRNA92a* has been reported to have a role in regulating proliferation of a number of cancer types [34]. For example, *miRNA92a* has been reported to be a critical regulator of the apoptosis pathway in glioblastoma and lymphoma. Specifically, *miRNA92a* has anti-apoptotic activity via inhibition of the pro-apoptotic protein BIM and inhibition of *miRNA92a* leads to induction of cell death [35,36]. In fact, down-regulation of *miRNA92* in human plasma is a novel marker for acute leukemia patients [37]. Moreover, *miRNA92a* was observed to promote tumor growth of osteosarcoma by targeting the PTEN/AKT signaling pathway [38]. Additionally, deregulation of *miRNA92a* expression has been implicated in hepatocellular carcinoma [39], and, in breast cancer, estrogen receptor expression was reported to be regulated by *miRNA92a* [40]. Other studies have also reported on the function of *miRNA92a* in CRC development. In one study, overexpression of *miRNA92a* correlated with tumor metastasis and poor prognosis in patients with CRC [41]. In a recent report, Alcantara and Garcia [42] found that *miRNA92a* promotes cell proliferation, migration and survival by directly targeting the tumor suppressor gene *NF2* in colorectal and lung cancer cells. Tsuchida et al. [26] found that increased expression of *miRNA92a* occurs in colonic adenomas and in CRCs, *miRNA92a* directly targets BMI and inhibits CRC cell apoptosis. In addition, *miRNA92a* inhibitor induces CRC cell apoptosis. In two other studies [43,44], *miRNA92a* in feces and plasma was found to serve as a biomarker for patients with colon tumors. Taken together, these reports indicate that *miRNA92a* has an important role in the pathological development of many cancer types, including CRC.

### 3.3. What Is the Role of miRNA92a in the Stem Cell Origin of Cancer?

MicroRNAs contribute to the progressive changes in gene expression that occur during development [45]. Indeed, expression patterns of many miRNAs are timing- and organ-specific, particularly miRNAs involved in the regulation of development. Notably, the *miRNA92a* family has an important role in regulating the development of mammalian organs including heart, lungs and immune system as well as the formation of blood vessels [35]. That *miRNA92a* has a critical role in development suggests that it regulates SCs during development [46]. For example, analyses of miRNA libraries have shown *miRNA92* to be expressed in mouse embryonic stem cells [47]. Such totipotent SCs represent the earliest stages of mammalian development because they are derived from the inner cells of the blastocyst. While miRNAs have a role in embryonic SCs and aberrant expression of miRNAs occurs in various cancers, their role in the SC origin of cancer is not very well understood.

Our findings herein show that *miRNA92a* downregulates *LRIG1* expression by targeting *LRIG1*′s 3′UTR region. We also observed that increasing *miRNA92a* levels increases CRC cell numbers and vice versa. Moreover, *miRNA92a* expression is upregulated in ALDEFLUOR-positive colonic CSCs. And, we found that ALDH-positive cells in CRCs do not express LRIG1 [48]. To understand the implications of our results indicating that expression of *miRNA92a* and *LRIG1* are inversely correlated in ALDH-positive CSCs, we need to understand how our findings relate to LRIG1′s known functional roles. Notably, LRIG1 is known as a pan-ERBB negative regulator and it is well established that LRIG1 promotes SC quiescence especially in epidermis and the GI tract system [33,49].

This raises the question: How does upregulated *miRNA92a* and reduced *LRIG1* affect the proliferative state of ALDH-positive colonic CSCs? We have recently studied the quiescence properties of human colonic crypt cells [50]. We found that several kinetic mechanisms regulate quiescence of normal crypt cells. And during CRC development, dysregulation of these kinetic mechanisms induces changes in crypt cell quiescence associated with the increased number of proliferative cells that occurs in colonic tumors. We also know that ALDH is a key component of the retinoid signaling pathway and ALDH-positive SCs directly differentiate into neuroendocrine cells [51]. Thus, we hypothesize that upregulated *miRNA92a*, by down-regulating *LRIG1*, decreases differentiation and increases proliferative potential of ALDH-positive CSCs. In this way, dysregulated expression of *miRNA92a* may contribute to CSC overpopulation that drives an increase in proliferative cells during CRC development and growth [3,4,5,52].

## 4. Materials and Methods

*Isolation of colonic epithelium*. The use of human clinical samples in this study was approved by the Thomas Jefferson University and Christiana Care Health System IRBs. Informed written consent was obtained from the subjects (wherever necessary). The patient studies were conducted in accordance with the following ethical guidelines: Declaration of Helsinki, International Ethical Guidelines for Biomedical Research Involving Human Subjects (CIOMS), Belmont Report, and U.S. Common Rule. Colon tissues removed from patients during surgery were collected during or immediately after surgery and crypts were isolated as we previously described [53,54,55]. All tissue samples (n = 5) were patient-matched (i.e., tumor and normal tissues were derived from the same patient).

*Isolation of Crypts.* Normal and tumor tissue were washed three times in sterile PBS. The normal tissue was carefully cleaned of the muscle layer using clean forceps and surgical scissors. It was then placed in 3.0 mM solution of EDTA containing DTT and incubated on ice for 60–90 min. The crypts were separated from the submucosa by rigorous shaking of the tissue in solution for five minutes. The isolated crypts were washed with sterile PBS thrice.

*Dissociation of Normal and Tumor Tissue to Single Cells*. Single cell dissociation of normal epithelium was achieved by treating the crypts with collagenase IV (Worthington Inc., Lakewood, NJ) solution in HBSS (10 mg/mL) and DNase I (Worthington Inc., Lakewood, NJ) for 60–90 min at 37 °C. Tumor tissue was cut into small pieces using a sterile scalpel and incubated in collagenase/hyaluronidase and DNase I solution in HBSS to achieve enzymatic digestion of the tissue to single cells. Cells from both normal and tumor tissue were washed thrice with HBSS.

*Identification of ALDH-positive Colonic Stem Cells in Fresh Viable Tissue Samples*. To detect ALDH activity in the bottom of fresh normal isolated crypts, single dissociated crypts were obtained after 60 min of dissociation from normal epithelium. These isolated colonic crypts, as well as fresh dissociated normal and tumor cells, were then subjected to ALDEFLUOR assay in the presence or absence of the DEAB inhibitor of ALDH activity. The detection of green fluorescence staining, which indicates high ALDH activity, was analyzed using a Zeiss Epi-fluorescence microscope using the 10X objective. The cells identified with high ALDH activity were then isolated using flow cytometry as described below.

*Overall Strategy*. Our study analyzed all the patient samples that were processed and produced RNA that passed quality control for profiling. Briefly, fresh tissue tumor/normal tissue pairs were obtained from five patients for processing and analysis. Specifically, we sorted using FACS, four different sorted cell populations from each of the patients for profiling: (1) PI-negative/ALDH-positive from normal tissue, (2) PI-negative/ALDH-negative from normal tissue, (3) EPCAM-positive/ALDH-positive from tumor tissue, and (4) EPCAM-positive/ALDH-negative from tumor tissue. We then isolated RNA from each of these 20 purified cell populations and only those RNA isolates that passed rigorous quality control standards were then analyzed by miRNA profiling. The RNA samples from four patients passed quality control and were analyzed for expression of 800 miRNAs using Nanostring profiling. This analysis of these multiple samples gave 12,800 data points for statistical analysis.

Analysis of this data from multiple patients gives the combined pattern of miRNA expression across the profiling of RNA from all four different isolated cell populations from all four of the patients. Statistical analysis provided two data sets: (i) the top set of miRNAs that showed a substantial difference in expression (<0.1) between tumor ALDEFLUOR-positive stem cells and ALDEFLUOR-positive normal stem cells, and (ii) a sub-set that showed a statistically significant difference (*p* < 0.05) in expression. This sub-set of miRNAs was analyzed further to identify SC marker genes that are targeted by the miRNAs that are differentially expressed in tumor ALDEFLUOR-positive stem cells compared to ALDEFLUOR-positive normal stem cells. Specific details on methods are given below.

*ALDEFLUOR Assay, Propidium Iodide Staining and EpCAM Staining of Normal and Tumor cells*. ALDEFLUOR-positive cells were isolated as described previously [5,17,51,56]. The dissociated cells from both normal and tumor were washed thrice in sterile HBSS and then subjected to the ALDELFUOR Assay following the manufacturer’s protocol (STEMCELL Technologies Inc., BC, Canada). The cells were spun down at room temperature and resuspended in ALDEFLUOR buffer. The cell suspension was divided into three tubes: ALDEFLUOR only, ALDEFLUOR + DEAB (inhibitor) and Blank. The tubes were incubated in the dark at 37 °C for 25 min. After incubation, the cells were spun down once and resuspended in ALDEFLUOR buffer. The cells suspension was transferred to a FACS sample tube through a 50-micron filter. The samples were run on the BD FACSAria II using the FACSDiva Software. The gates were drawn to eliminate doublets and to include 0.1% in the positive gate of cells in the respective DEAB control and appropriate ALDEFLUOR positive samples. Normal dissociated tissue was stained for propidium iodide to identify live cells following the ALDEFLUOR staining. Cells were incubated in ALDEFLUOR buffer containing PI stain at a concentration of 1 μg/mL for 20 min at RT in the dark. Cells were washed once with ALDEFLUOR buffer and were stored on ice under further analysis. Tumor cells, after the ALDEFLUOR assay, were incubated with an APC-labeled EpCAM antibody (1:100, BioLegend Inc., San Diego, CA) for 1 h on ice in the dark. EpCAM staining was used to select colon carcinoma cells and exclude contaminating stromal cells from tumor tissues. The sorted cells were washed twice with ALDEFLUOR buffer and were stored on ice until they were subjected to FACS analysis.

*Cell Sorting by Flow Cytometry*. The 85-micron or the 100-micron nozzle was used for the sorting experiment. The stream was turned ON and was Cytometer setup and Tracking was done to calibrate the machine. Accudrop experiment was carried out to determine the delay between the drops prior to any sorting. A test sort was done to ensure proper collection of sample in the collection tubes. The dot plots were created and the cell populations were appropriately chosen to eliminate doublets out of the analysis. The blank sample was run through FACS initially. Voltage of FSC and SSC were adjusted if needed to set the cell population in the center of the first dot plot. Appropriate regions within the plots were selected so that only single cells could be sorted. The sample with the DEAB control was run through FACS and data was recorded. The ALDH alone (green only) and the PI alone (red only for normal) or EpCAM only (red only for tumor) samples were run to calculate compensation between the two fluorophores to eliminate cross talk in the analysis. Using the DEAB alone sample, the gates for the ALDEFLUOR negative and ALDEFLUOR positive were created in the dot plot for SSC vs FITC. Similarly, gates for additional controls (PI and EpCAM) were also created using the blank sample. For normal tissue, PI negative samples were regarded as alive and were gated for ALDEFLUOR positive or negative cells. For the tumor samples, EpCAM positive cells were selected and gated for ALDEFLUOR-positive and ALDEFLUOR-negative cells. After quality control, we obtained four different sorted samples from each pair: PI-negative/ALDH-positive and PI-negative/ALDH-negative from normal tissue and EPCAM-positive/ALDH-positive and EPCAM-positive/ALDH-negative from tumor tissue. These four pairs of sorted samples were used for isolation of RNA and analysis by miRNA profiling.

*Cell Lines*. The HT29 cell line was purchased from ATCC Inc., (Manassas, VA; year 2013; authenticated by the provider by cytogenetic analysis). Cells were grown in McCoys media (GIBCO) containing 10% FBS and 1% Penicillin/Streptomycin. The cells were grown in 5% CO_2_ and 95% air at 37 °C. All the experiments were carried out in triplicate and within 10 passages after being thawed. The cells were routinely tested for mycoplasma by Universal mycoplasma detection kit by ATCC.

*RNA Isolation*. RNA isolations were performed according to the manufacturer’s protocol using the Trizol Reagent (Life Technologies Inc., Carlsbad, CA). One mL of Trizol was added per million cells and incubated at 4 °C for ten minutes. Chloroform (0.2mL per ml of Trizol used) was added to the cells and the solution was mixed vigorously for 15 s. The tubes were placed on ice for two min and then spun at 12,000 rpm at 4 °C for 15 min. Following centrifugation, the upper clear layer was carefully removed from each sample and transferred to a new tube. RNA was precipitated by adding isopropanol (0.5 mL per 1 mL of Trizol added) to the tube, followed by incubation for 10 min on ice. Spinning down the solution at 12,000 rpm for ten minutes at 4 °C pelleted the precipitated RNA. The supernatant was discarded and the RNA was washed twice by spinning down the pellet in 1 mL of ice cold 70% ethanol per ml of Trizol used. The pellet was allowed to air dry and was dissolved in nuclease free water.

*RNA Quantification*. RNA quantification was done by determining the absorbance of samples at two wavelengths (260 and 280 nm) using the plate reader (TECAN) and the Nanoquant plate. Two μl of sterile water was used for blanking the instrument, followed by 2 μL of the RNA sample. The concentration values provided by the instrument were taken in triplicates and averages were calculated for each RNA sample.

*MicroRNA Analysis*. The RNA samples were concentrated to get a minimum concentration of 33 ng/μL by using 3.0 M Sodium acetate and glycogen as a carrier molecule. The samples were sent to Nanostring Technologies Inc. (Seattle, WA) for profiling. Nanostring Technologies made use of a unique set of color-coded fluorescent probes, which is hybridized to mature miRNA with the help of a linker molecule. This approach has the advantage of quantitation of miRNA levels in a cell population and requires as little as 150 ng of total RNA per sample.

The results were presented as raw counts for each of the miRNAs as well as several negative and positive controls. This data was then imported to NSolver 4.0 software. The software takes the average of the whole data set and ranks the miRNAs with the highest positive and negative values from the average. Each measured miRNA species in each sample is determined to present or absent by comparison to the internal negative controls for that sample. Counts corresponding to “present” miRNAs are then normalized using a set of housekeeping genes, where the geometric mean of housekeeping genes is calculated. The software then estimates the arithmetic mean of the geometric means for all sample lanes. It derives the lane specific normalization factor by dividing the arithmetic mean with geometric mean of each lane and multiples each count by this factor to give the normalized value. These normalized counts are then compared between samples to get fold changes between miRNAs expression. Relative fold change was calculated for each miRNA. This was achieved by dividing the normalized count for a miRNA in the ALDEFLUOR positive sample by the count in the ALDEFLUOR negative counterpart. Candidate miRNAs, which showed differential expression in tumor ALDEFLUOR positive cells as compared to normal ALDEFLUOR positive cells, were selected for further analysis. The heatmap in Figure 2 is representative of log2 fold changes of normalized value of each miRNAs, centered and normalized to the mean of each lane. The miRNAs represented in Figure 2 have a cut off *p* value significance of 0.1 or lower.

*Prediction and analysis of gene targets for miRNAs*. The predicted target transcripts of miRNAs differentially expressed in CRC SCs were used to identify predicted target genes. Genome-wide miRNA-mRNA target prediction was done using rna22 and TARGETSCAN by computationally seeking “hits” on the 3’UTRs of all known transcripts as described [17].

*Quantitative RT-PCR miRNA Assay*. Quantitative RT-PCR was performed using a hydrolysis probe(s)-type assay, RT-PCR kit, and miRNA-specific probes according to the manufacturer’s instructions (Applied Biosystems, Foster City, CA & Life Technologies Inc., Carlsbad, CA) as follows. Twenty nanograms of RNA were used in a final volume of 5 uL. Primer for the miRNA or U6 Control (3 μL), dNTPs (0.15 μL), 10X PCR buffer (1.5 μL), RNAse Inhibitor (0.19 μL), RT enzyme (1.0 μL) and water were added to make a final volume of 15 μl. The samples were mixed gently using a sterile pipette and spun down briefly. The samples were placed in the thermocycler and cDNA was synthesized using the following temperature conditions: 16 °C for 30 min, 42 °C for 30 min, 85 °C for 5 min and 4 °C for 1 h. The cDNA (1.33 μL of RT product) was used to prepare the RT-PCR reaction, which also contained 10ul of 2X PCR master mix (Applied Biosystems, no AmpErase UNG), the primers provided in the kit and water to a final volume of 20 uL. The samples were prepared in triplicates and added to the 96 well thermocycler plate (VWR Inc., Radnor, PA). The RT-PCR was run using Applied Biosystems 7500 fast PCR machine for 95 °C for 10 min followed by 40 cycles of 95 °C for 15 s and 60 °C for 1 min. The Ct value obtained was used to calculate the average Ct for each sample. The fold change in expression of miRNA in treatment versus control samples was calculated using the formula 2^–(delta Ct (treatment) − delta Ct (control)^. Four replicates were run per tumor/normal sample pair. Any value greater than 2-fold change was regarded as significant. Statistical analysis was done by Student’s T-test: Paired Two Sample for Means.

*Transient Transfection. MicroRNA92a* antimir and precursor siRNA molecules were purchased from Ambion, Life Technologies. The amount of siRNA and transfection reagent (Lipofectamine 2000, Invitrogen) was standardized for HT29 using a GFP coupled scrambled siRNA from DHARMACON Inc., (Pittsburgh, PA). The cell lines were transfected in a 12 well or a 6 well plate and media was changed 24 h post transfection. The increase or decrease of the miRNA levels was tested by the miRNA RT-PCR Assay. The cells were analyzed 24–48 h post transfection.

*Proliferation Assay*. Cells were plated in triplicate per condition in 24-well plates and the initial count was regarded as day 0. Seventy-two hours after treatment, cells were trypsinized, counted and analyzed for viability by trypan blue dye exclusion using a Countess Cell counter (Invitrogen). The enumerated cell numbers represented cell proliferation for day 3. Three independent sets of experiment were carried out for each condition. Normalized fold change in cell count of HT29 cells with increased and decreased levels of *miRNA92a* was calculated by dividing the cell count of treatment group as compared to control groups.

*Luciferase Assay*. Plasmids expressing luciferase and the 3′UTR of the predicted target gene, *LRIG1*, was purchased from GeneCopoeia. Thirty-thousand HT29 cells were plated in a 96 well plate and 24 h later co-transfected with the luciferase vector and *miRNA92a* precursor and precursor control molecules using Lipofectamine 2000. The media was changed 24 h post-transfection and the cells were used for dual-color luciferase assay (Promega) and luminescence was recorded using the TECAN plate reader.

*Statistical Analysis*. An Unpaired *t*-test was used to determine significance between the treatment group and the appropriate controls. All the values obtained with a *p*-value less than 0.05 were considered to be statistically significant and the corresponding miRNA a candidate for further experimental validation.

## 5. Conclusions

Our goal was to determine if dysregulation of miRNAs is involved in the SC origin of CRC. Accordingly, we used Nanostring-based miRNA expression profiling of ALDH-positive SC isolated from normal colonic epithelium and CRC tissue. We found that *miRNA92a* is selectively expressed in normal SCs and is aberrantly expressed in CRC SCs. Our further analysis revealed that *miRNA92a* is a regulator of the *LRIG1* stem cell gene. As discussed above, LRIG1 is a tumor suppressor gene, which is involved in the inhibition of EGFR signaling. Indeed, LRIG1 was identified as a robust marker for colonic SCs in humans as well as in mice [28,49]. Our findings indicate that increased levels of *miRNA92a* diminish the tumor suppressive role of LRIG1 in the ALDEFLUOR-positive CSC population. Taken together, our results suggest that *miRNA92a* is a critical contributor to the SC origin of CRC.

## Supplementary Materials

The following are available online at https://www.mdpi.com/1422-0067/21/8/2804/s1. Table S1. List of matched normal and tumor colon samples sorted for ALDEFLUOR positive cells. Table S2. List of Differentially Expressed miRNAs shown in Figure 2B Heatmap. For Normal & CRC, values are ratio of ALDH+/ALDH−. (all with *p* < 0.1, * *p* < 0.05). Figure S1 The heat map represents the normalized log2 fold change from the mean across the four samples for each miRNA. Figure S2. Expression of the four miRNAs (*miRNA92a, miRNA200c, miRNA93, miRNA20a*) identified as having significantly (*p* < 0.05) altered expression in ALDEFLUOR-positive tumor CSCs as compared to ALDEFLUOR-positive normal SCs.
