# Supplementary material for: MicroRNA Expression Profiling of Normal and Malignant Human Colonic Stem Cells Identifies miRNA92a as a Regulator of the LRIG1 Stem Cell Gene
Source: Int J Mol Sci. 2020 Apr 17;21(8):2804. doi: 10.3390/ijms21082804 (PMC7216254; doi:10.3390/ijms21082804)
Supplement: Supplementary file 1 [file ijms-21-02804-s001.pdf]

Supplementary Material

Table S1. List of matched normal and tumor colon samples sorted for ALDEFLUOR positive cells.		
**Patient**	**Proportion of ALDEFLUOR-positive Cells from Normal Colonic Epithelium**	**Proportion of ALDEFLUOR-positive Cells from Colon Carcinomas**
1	4.0%	1.2%
2	6.0%	1.9%
3	2.8%	2.4%
4	1.8%	1.2%
5	4.2%	1.0%

Table S2. List of Differentially Expressed miRNAs shown in Figure 2B Heatmap.
For Normal & CRC, values are ratio of ALDH+/ALDH–. (all with $p < 0.1$, *$p < 0.05$)

MicroRNA	Normal	CRC	CRC/Normal
hsa-miR-302c	0.04	1.16	31.345
hsa-miR-548j	0.13	1.31	9.746
hsa-miR-184	0.23	2.11	9.224
hsa-miR-548h	0.23	2.11	9.224
*hsa-miR-20a+hsa-miR-20b	0.35	3.20	9.088
hsa-miR-648	0.38	2.03	5.316
hsa-miR-486-3p	0.38	2.03	5.316
hsa-miR-539	0.28	1.21	4.300
*hsa-miR-92a	0.52	1.58	3.069
hsa-miR-1254	0.46	1.34	2.896
hsa-miR-675	0.69	1.84	2.679
hsa-miR-145	0.50	1.24	2.478
hsa-miR-623	0.50	1.24	2.478
hsa-miR-595	0.46	1.10	2.374
hsa-miR-615-5p	0.38	0.86	2.285
hsa-miR-125b	0.53	1.15	2.167
hsa-miR-100	0.54	1.15	2.126
hsa-miR-208b	0.63	1.24	1.952
hsa-miR-1915	0.21	0.38	1.775
hsa-miR-181b+hsa-miR-181d	0.22	0.38	1.719
hsa-miR-363	0.19	0.30	1.559
hsa-miR-617	0.99	1.52	1.527
hsa-miR-409-3p	0.67	1.00	1.493
hsa-miR-584	0.75	1.03	1.369
hsa-miR-1206	0.38	0.47	1.255
hsa-miR-106a+hsa-miR-17	0.72	0.83	1.159
hsa-let-7a	0.84	0.88	1.051
hsa-miR-769-5p	0.50	0.52	1.048
hsa-miR-410	0.41	0.43	1.040
hsa-miR-654-3p	0.63	0.63	0.994
hsa-miR-34b	0.50	0.47	0.936
hsa-miR-134	0.50	0.47	0.936
hsa-miR-744	0.63	0.56	0.880
hsa-miR-517c+hsa-miR-519a	0.73	0.61	0.833
hsa-miR-548f	1.02	0.80	0.788
hsa-miR-2276	1.25	0.98	0.785
hsa-let-7b	1.04	0.77	0.745
hsa-let-7g	0.78	0.53	0.675
hsa-miR-499-5p	0.61	0.39	0.644
hsa-miR-106b	1.08	0.58	0.535
hsa-miR-30c	1.56	0.79	0.509
hsa-miR-107	0.86	0.40	0.469
hsa-miR-133a	0.71	0.31	0.439
hsa-miR-514	1.03	0.43	0.420
hsa-miR-16	0.65	0.20	0.313
*hsa-miR-200c	1.04	0.27	0.255
hsa-miR-521	1.60	0.31	0.195
hsa-miR-938	1.60	0.31	0.195
*hsa-miR-93	87.19	0.27	0.003

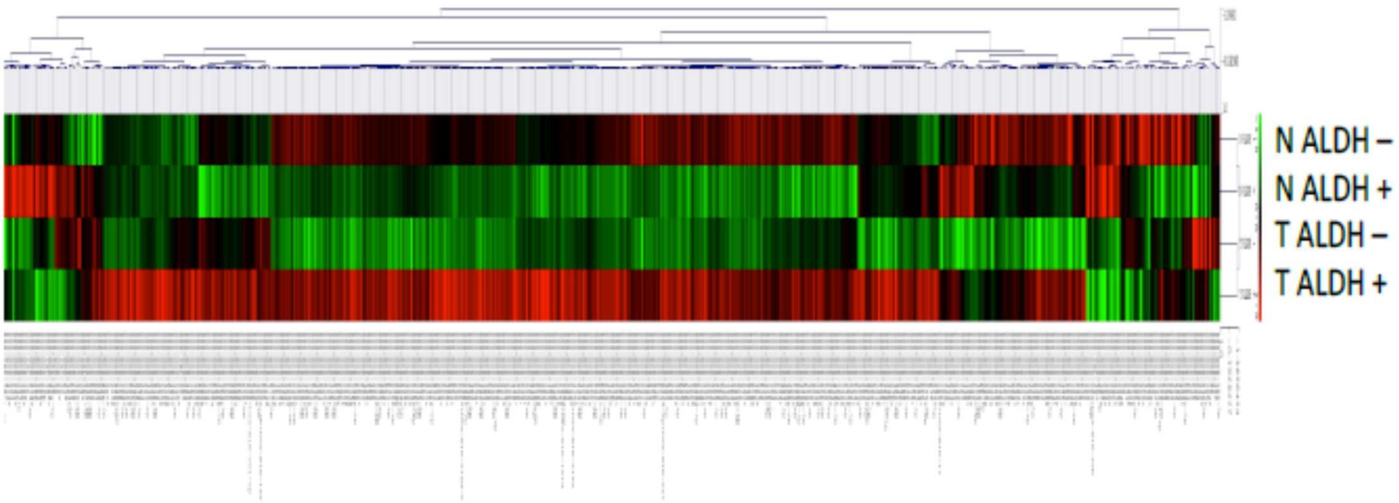

Figure S1 The heat map represents the normalized log2 fold change from the mean across the four samples for each miRNA. Red represents up-regulation and green represents down-regulation. This figure illustrates the <u>patterns</u> that are seen when a large set of miRNAs is surveyed (n = 800); it is not meant to show details, which is why the horizontal axis is not legible.). It is a representative experiment involving the four sorted cell populations isolated from matched fresh normal and tumor tissue from one CRC patient.

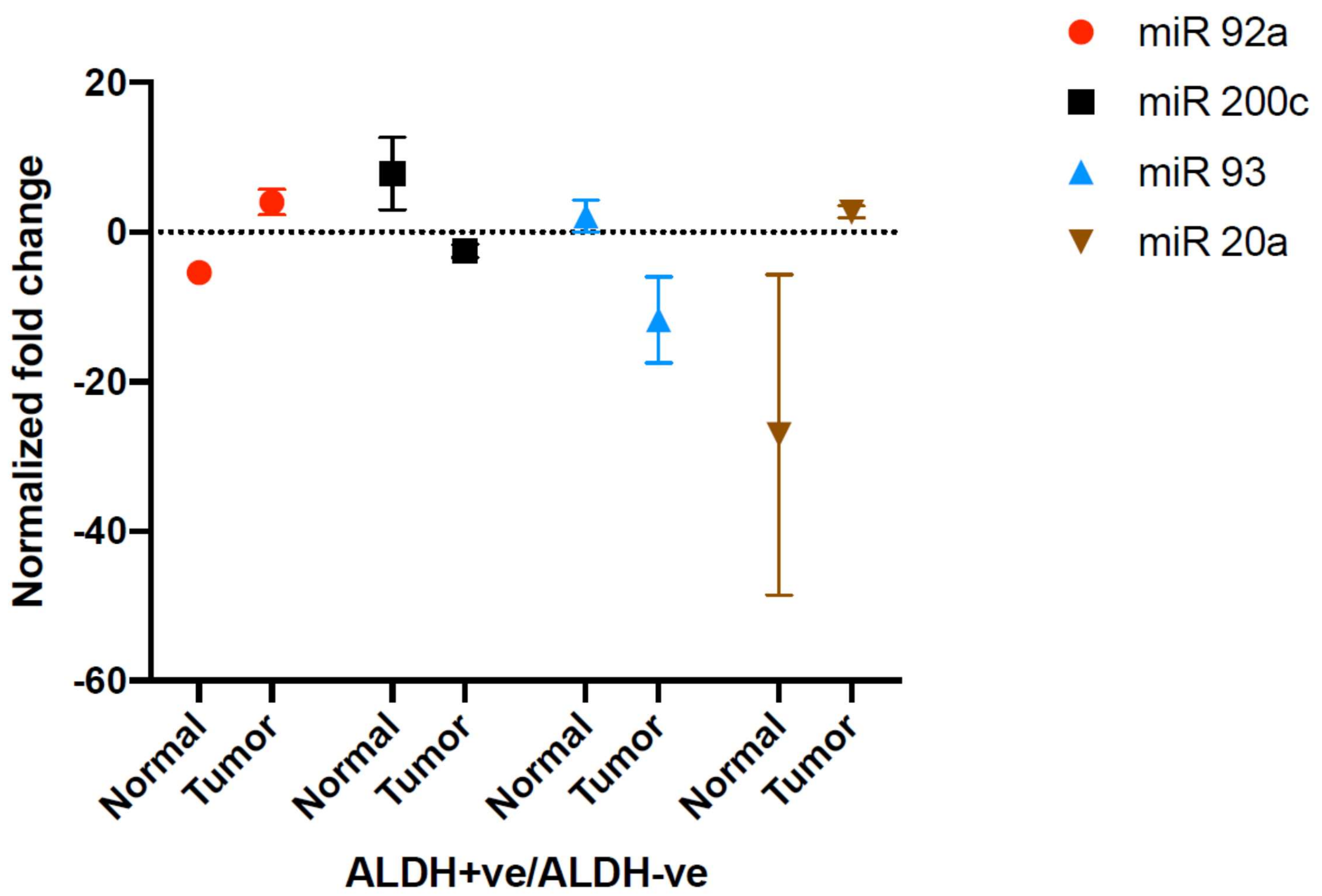

Figure S2. Expression of the four miRNAs (miRNA92a, miRNA200c, miRNA93, miRNA20a) identified as having significantly ($p<0.05$) altered expression in ALDEFLUOR-positive tumor CSCs as compared to ALDEFLUOR-positive normal SCs
